# Controlled experiments to explore the use of a multi-tissue approach to characterizing stress in wild-caught Pacific halibut (*Hippoglossus stenolepis*)

**DOI:** 10.1093/conphys/coab001

**Published:** 2021-02-05

**Authors:** Anita C Kroska, Nathan Wolf, Josep V Planas, Matthew R Baker, T Scott Smeltz, Bradley P Harris

**Affiliations:** 1 Fisheries, Aquatic Science, and Technology Laboratory, Alaska Pacific University, 4101 University Dr., Anchorage, AK 99508, USA; 2 International Pacific Halibut Commission, 2320 W Commodore Way, Seattle, WA 98199, USA; 3 North Pacific Research Board, 1007 W 3rd Ave #100, Anchorage, AK 99501, USA

**Keywords:** Cortisol, dynamics, epidermal mucus, Pacific halibut, plasma

## Abstract

The integration of multiple tissues in physiological and ecological analyses can enhance methodological approaches, increase applications for data and extend interpretation of results. Previous investigations of the stress response in fish have focused primarily on cortisol levels in a single matrix—blood plasma—which confines interpretations of cortisol levels to a short temporal frame. Epidermal mucus has been proposed as an alternative or complement to plasma that may provide a view to cortisol levels over a different temporal window allowing comparative assessment. Here, we explore the potential for multi-tissue cortisol analysis using both plasma and epidermal mucus in Pacific halibut (*Hippoglossus stenolepis*). The relative timing at which cortisol increased and decreased in the two matrices as well as cortisol concentrations at estimated peak levels were compared in two trials after (i) inducing cortisol synthesis by adrenocorticotropic hormone (ACTH_1–24_) administration and (ii) inducing cortisol elimination using cortisol (hydrocortisone, 98%) injection. The ACTH treatment elicited a peak plasma cortisol response approximately 12 hours post-injection, while mucus cortisol concentrations peaked later at approximately 62 hours post-injection. Exogenous cortisol treatments suggested relatively little transfer of cortisol from plasma to mucus, potentially reflecting differential effects of endogenous and exogenous cortisol. Our results suggest the potential utility of mucus as a sampling matrix that provides an extended window for detection of the stress response as compared to plasma. Results also suggest the utility of a multi-tissue approach to cortisol analysis with potential applications to applied fisheries research. Increased understanding of the relative scale of the cortisol response to stress (e.g. capture) will allow researchers and managers to better interpret the physiological condition and survival outcome of fish subjected to regulatory discard.

## Teaser

We provide an experimentally derived comparison of the kinetics of cortisol in Pacific halibut plasma and epidermal mucus. Our results suggest the potential for a multi-tissue approach to cortisol analysis that provides two distinct temporal windows without the need for repeated sampling when evaluating stress responses.

## Introduction

Multi-tissue approaches to commonly used analyses in physiology and ecology have allowed for increased application, interpretability and resolution. For example, multi-tissue analyses of stable isotopes have revealed unique temporal windows through which changes in resource use can be determined and traced (e.g. Carleton *et al.*, 2008). Likewise, multi-tissue comparisons of gene expression have uncovered commonalities in regulation across tissues or matrices, thus aiding our understanding of phenotypic differentiation (e.g. Petretto *et al.*, 2010). Previous assessments of stress hormones in fish have principally focused on analysis of a single matrix, primarily blood plasma (Wendelaar Bonga, 1997; Mommsen *et al.*, 1999; Baker *et al.*, 2013a). This is, presumably, due to the rapid rate at which plasma cortisol concentrations increase following the introduction of a stressor (Haukenes and Buck, 2006) as a result of the role of blood in transporting endocrine molecules like cortisol. Upon detecting a stressor, the central nervous system signals the hypothalamus to produce corticotropin-releasing hormone (CRH) that, in turn, stimulates the release of adrenocorticotropic hormone (ACTH) from the anterior pituitary. ACTH then stimulates the synthesis and release of cortisol (in addition to other glucocorticoids) from the interrenal tissue into the blood (Romero and Butler, 2007; Touma and Palme, 2005; Wendelaar Bonga, 1997). During this acute stress response, cortisol and other glucocorticoids are distributed to target tissues, where they bind to intracellular receptors to regulate gene transcription and translation, resulting in increased gluconeogenesis, altered behaviour and the downregulation of physiological processes not necessary to respond to the stressor (e.g. growth, reproductive activity, immune response; (Das *et al.*, 2018; Romero and Butler, 2007). Following the subsidence of the stressor, circulating cortisol is metabolized, and normal physiological processes are resumed (Mommsen *et al.*, 1999).

The rapid response rate of plasma cortisol levels, and consequent ability of plasma cortisol measurements to reflect levels at or near the time of a stressor, explains much of the prevalence of this technique in the literature (Pankhurst, 2011). However, the short temporal frame of inference imposed by this quick response rate potentially limits the use of plasma as a sampling matrix for many investigations. For example, for organisms in natural settings, the capture and sampling processes may themselves be reflected in the plasma cortisol levels, thereby resulting in potential overestimation of post-stressor cortisol levels (Millspaugh and Washburn, 2004; Romero and Reed, 2005; Touma and Palme, 2005; Bertotto *et al.*, 2010). Furthermore, practical considerations may limit the applicability of sampling plasma in many field settings, such as on moving vessels, in species for which anaesthesia is required, or where large sample sizes are required. By employing a second sampling matrix that is easily collected and displays a relatively delayed response rate, we can (i) expand the range of field conditions in which cortisol measurements can be applied, (ii) extend the temporal window of inference over which cortisol measurements can be interpreted and (iii) support comparative analysis of cortisol levels between matrices; thereby allowing for investigations of fluctuations in cortisol over time without the need for repeated sampling.

Previous studies investigating or employing the use of alternative matrices for measuring cortisol in vertebrates have focused on scales (Aerts *et al.*, 2015), structural tissues (e.g. muscle; Simontacchi et al., 2008) and faeces (Ashley *et al.*, 2011; Cao *et al.*, 2017), to name a few. Despite their success in assessing cortisol concentrations, these matrices all involve either some amount of invasive sampling or are more or less suitable for marine organisms (e.g. locating and sampling excreta) (Bertotto *et al.*, 2010; Rolland *et al.*, 2007; Wasser *et al.*, 2017). Epidermal mucus has been identified as an alternative matrix that can be collected quickly, easily and in a relatively non-invasive fashion from marine organisms. Bertotto et al. (2010) examined cortisol levels in plasma and mucus from three different fish species (European sea bass (*Dicentrarchus labrax*), common carp (*Cyprinus carpio*) and rainbow trout (*Oncorhynchus mykiss*)) in aquaculture settings. Following the introduction of a physical stressor, the authors observed significant increases in cortisol levels in both plasma and mucus and concluded that mucus cortisol is a viable candidate for measuring stress in fish. While Bertotto *et al.*’s (2010) investigation did not examine the relative response rates of plasma and epidermal mucus cortisol levels to stressors, the relatively indirect route by which cortisol reaches epidermal mucus (i.e. after passing from target tissues, glucocorticoids, such as cortisol, are excreted through various pathways including the goblet cells that secrete mucus granules; Shephard, 1994) suggests that the response rate of epidermal mucus cortisol levels to the introduction of a stressor will be slower than that for plasma. Consequently, epidermal mucus has the potential to provide a broader temporal window through which cortisol levels can be compared to plasma.

Before epidermal mucus cortisol concentration analyses can be applied in field settings, however, both the timing and magnitude with which cortisol is reflected in epidermal mucus following an acute stressor must be ascertained in a manner that allows for direct comparison to blood plasma. To permit direct comparison, both the timing of cortisol synthesis and distribution and the timing of cortisol elimination from these individual matrices must be better understood. Exogenous ACTH injections have previously been used to induce an acute stress response to determine both resting and stressed plasma cortisol levels in fishes, including sailfin mollies (*Poecilia latipinna*; Kim *et al.*, 2018) and white sturgeon (*Acipenser transmontanus*; Belanger *et al.*, 2001). Likewise, exogenous cortisol injections have been used to measure cortisol absorption and elimination timing in the plasma of Atlantic salmon (*Salmo salar* L.; Espelid *et al.*, 1996).

In addition to our interest in increasing understanding of cortisol kinetics in large marine fishes, and concomitantly their hypothalamic–pituitary–interrenal (HPI) axis and stress response, our explorations were guided by the applied question of whether an acute stressor (such as capture) could be detected initially in plasma and subsequently in mucus, and whether comparative analyses of cortisol measurements in these two matrices could be used to improve post-release survival estimates of Pacific halibut caught by fishing gear. Currently, post-release survival is estimated using qualitative visual examinations and is based on no physiological information (AFSC, 2017). Little is known about the Pacific halibut stress response, especially in regard to comparative analyses across possible sampling matrices. We suggest that adding multi-tissue cortisol analyses would enable measurements of stress levels before and after capture during one sampling event; thereby allowing for both detection of physical and/or physiological trauma that cannot be revealed by visual examination (Baker and Vynne, 2014) resulting in a better characterization of the effects of fishing-induced stress on Pacific halibut. Understanding the relative scale of the magnitude and timing of the cortisol response to stress will allow researchers and managers to begin to interpret the impacts of fishing on the fish’s physiological condition and subsequent survival of discarded fish.

Here, we report the results of an experiment designed to examine cortisol dynamics from a multi-tissue perspective in Pacific halibut (*Hippoglossus stenolepis*). More specifically, we examined the relative timing and magnitudes of increases and decreases in blood plasma and epidermal mucus cortisol levels after (i) inducing cortisol synthesis and distribution using ACTH injection and (ii) inducing cortisol elimination using cortisol injection.

## Materials and methods

### Fish collection and housing

Capture, holding and experimental activities were authorized under Alaska Department of Fish and Game Aquatic Resource Permit No. CF-18-089, International Pacific Halibut Commission Permit No. EL2018049, and approved by the Alaska Pacific University Institutional Review Board.

Pacific halibut (*n =* 20) were collected by angling in September 2018 from the mouth of Resurrection Bay near Seward, Alaska (59.80° N, 149.23° W). All fish had fork lengths between 50 and 79 cm ($\overline{x} \pm sd$; 62.2 $\pm$ 4.1 cm). This size range was selected to minimize potential variations in cortisol response due to size (Barcellos *et al.*, 2012) and to represent the most common lengths of fish discarded in commercial halibut fisheries (81.3 cm minimum size limit; IPHC, 2017). Blood samples (*n =* 20*)* were obtained immediately after capture by ventral caudal puncture using 3-ml BD syringes with fine gauge needles (BD Luer-lok tip, 23 g x 1in needle; Becton, Dickinson and Company, Franklin Lakes, NJ) and stored in 4-ml heparinized vacutainer tubes (Grenier Bio-One Lithium Heparin Vacuette Tubes) on ice for no more than 30 minutes before separating via centrifugation at 1500 rpm for 30 mins. Plasma aliquots were stored at −80°C.

All fish were PIT tagged (12 mm; Biomark Inc., Boise, ID), and housed at the University of Alaska Fairbanks, Seward Marine Center (Seward, AK), where they were randomly assigned to one of four 6-ft diameter fiberglass tanks (~1468 L; 5 fish/tank) provisioned with continuous flow-through seawater on a 12:12 h photoperiod. Seawater was filtered, aerated, and held at ambient local water temperatures. Fish were fed three times per week *ad libitum* on an evenly mixed diet of herring and squid.

Fish were left undisturbed except for feeding and tank cleaning for an acclimation period of approximately 6 weeks after initial capture. This time period was selected to exceed Haukenes and Buck’s (2006) observations of elevated plasma cortisol levels in Pacific halibut ten days after the introduction of a stressor, and the time after which Pacific halibut exposed to capture stress resumed feeding, as reported by Davis and Olla (2001).

### ACTH administration

Following the initial acclimation period, treatment groups (ACTH injection or control injection) were randomly assigned at the tank level. Fish in three tanks (3 * 5 fish/tank; *n =* 15) each received a single 1.0 ml intraperitoneal injection of an ACTH solution (5.0 μM ACTH_1–24_, A0298; Sigma Aldrich; rehydrated with Ringer’s solution) following Belanger *et al.* (2001). Fish in one tank (*n* = 5) received a control treatment of a single 1.0 ml intraperitoneal injection of Ringer’s solution (Ringer’s tablets, 96724; Sigma-Aldrich). Plasma and epidermal mucus samples were collected from one fish from each tank (i.e. 3 fish for the ACTH injection treatment and 1 fish for the control treatment) at each of ten sampling times (*t*): 0, 2, 4, 8, 12, 24, 36, 48, 72 and 84 hours post-injection. Because of the limited number of fish available, each fish was sampled twice during the experimental period—once between 0 and 12 hours and again between 24 and 84 hours, always allowing at least 24 hours for recovery between repeated samplings. For example, a fish sampled 4 hours after injection would not be sampled again until hour 36, allowing at least 24 hours between handling events. Dividers were placed in the tanks to distinguish between sampled and non-sampled fish during the trials. Plasma sampling was conducted using the methods described above, and mucus samples (≈0.5 ml) were collected following Campbell (2015). Briefly, the long side of a glass microscope slide was gently scraped along the dorsal epidermal surface of the posterior lateral line on the ocular (right-eyed) side of the flatfish, stopping prior to the insertion of the caudal fin. Care was taken to avoid possible areas of contamination, and to avoid collecting scales or debris. Mucus samples were stored in individual 50 ml plastic tubes at −80°C.

### Cortisol administration

Following the ACTH trial, fish were left undisturbed for ≥30 days to reacclimatize. Following this period, treatment groups (cortisol injection or control injection) were randomly assigned at the tank level. Fish in two tanks (2 * 5 fish/tank; *n* = 10) each received a single 1.0 ml intraperitoneal injection of a cortisol solution. Cortisol preparation and dosage methods were modified from Espelid *et al.* (1996). Briefly, cortisol (ACROS Organics hydrocortisone, 98%, AC352450010) was dissolved in pure ethyl alcohol (200 proof, E7023; Sigma-Aldrich) to create a stock solution, and further diluted in Ringer’s solution to a final concentration of 450 μg of cortisol/1 ml of Ringer’s. This dosage was based on 0.1 μg/g of fish and an assumed body mass of 4.5 kg. Fish were not weighed to avoid disturbing them during the acclimation periods. Body mass estimates were based on average Pacific halibut length/weight ratios determined by the IPHC (2018). Fish in the two control tanks (2 * 5 fish/tank; *n* = 10) each received a single 1.0 ml intraperitoneal injection of Ringer’s solution. Plasma and mucus sampling were conducted using the protocols described for the ACTH injection experiment.

### Cortisol extraction and analysis

Cortisol concentrations in plasma and mucus were measured using enzyme-linked immunosorbent assay kits calibrated for plasma and saliva matrices, respectively (Calbiotech ELISA kits; CO368S, CO116S, respectively) following the manufacturer’s procedures. Mucus samples were processed following a modified version of the methods described in Guardiola *et al*. (2016). Briefly, thawed mucus samples were vortexed with filtered, sterilized seawater at a volume ratio of 1:1, mechanically mixed with a metal micro spatula to further homogenize the sample, and then vortexed again. Samples were then centrifuged at 1500 rpm for 5 min, and the supernatant was stored at −80°C for analysis. Plasma and mucus supernatant aliquots were serially diluted prior to measurement. Parallelism with the standard curve for each matrix extract was confirmed. Samples were analysed in duplicate, and plates were read at 450 nm in a microplate reader (Fisherbrand accuSkan FC, Fisher Scientific). Plasma inter- and intra-assay coefficients of variation were 3.96% and 10.95%, respectively. Mucus inter- and intra-assay coefficients of variation were 7.94% and 12.66%, respectively.

### Statistical analysis

To estimate the distribution and elimination timing of cortisol synthesis and relative magnitudes of cortisol concentration among treatments and matrices, we modelled cortisol concentration (ng/ml) as a cubic function of time after injection (hours). A cubic function was used as a heuristic model to capture a cortisol peak followed by an elimination stage that results in a local minimal cortisol concentration. Model parameters were estimated using a Bayesian approach to better estimate uncertainty with small sample sizes, correlated parameters and derived parameters. The intention, and benefit, of these statistical models was the ability to use the small sample size to make generalized inferences about the Pacific halibut stress response. A measured peak in cortisol could also be considered an estimated peak, as it reflects only a few individuals and the temporal resolution of the sampling. When developing a sample design, it can be difficult to develop the necessary temporal resolution of sampling to discern whether the maximum or minimum value of a parameter has been adequately captured. A peak in measured cortisol may just be the maximum value captured during the time window for that individual, when the true peak may have occurred between sampling times. Furthermore, utilizing a Bayesian framework allowed for the quantification of the uncertainty surrounding estimated maxima and minima. All models were fit using uninformed priors. Posterior densities of parameters were estimated using three Markov Chain Monte Carlo (MCMC) simulations (100 000 iterations), thinning to every third sample, and removing the first 100 samples to burn-in the model. MCMC simulations were run using JAGS software (Plummer, 2003) via an R interface (*rjags*). Model convergence was verified by visual inspection of the traceplots and ensuring that Rhat was <1.1 (Kery, 2010). Additionally, MCMC parameter estimates that did not meet the following *a priori* constraints were removed: (i) the local maximum of the estimated cubic function was located at *t* > 0, and (ii) cortisol estimates over the sampling window were all positive. All analyses were conducted using the R statistical software (v. 3.6.0, R Core Development Team).

**Figure 1:**
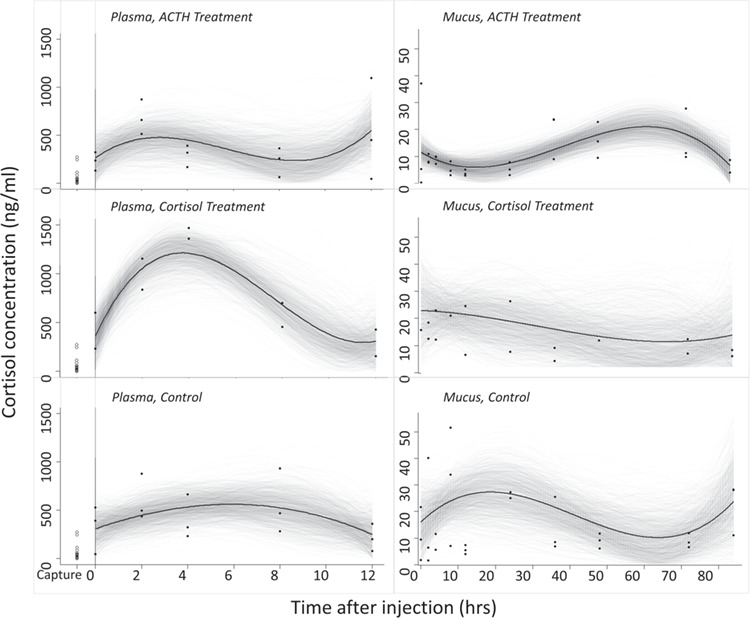
ACTH and cortisol trials Bayesian model outputs (raw data included as solid dots). Each column of plots represents a tissue (plasma, left; mucus, right), and each row represents a treatment (ACTH, top; cortisol, middle; control group, bottom). The grey curves represent separate model outputs, and the solid black curve denotes the mean model output. Note the differences in the x- and y-axes between plasma and mucus plots. ‘Capture’ refers to measured plasma cortisol concentrations from the time of capture. Plasma and mucus control plots include data pooled by tissue from both trials.

**Figure 2:**
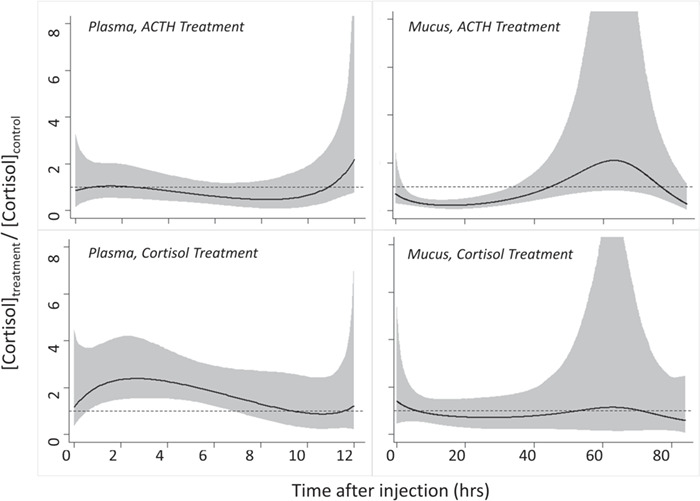
Treatment curves for each tissue (Figure 1) were divided by the corresponding control curve (e.g. plasma cortisol curves from ACTH treatment divided by control group) to demonstrate the proportional difference between the treatment and control groups, based on Bayesian model outputs. The mean curve (denoted by the solid black line) is plotted with a 90% credible interval band (the grey polygon area around each mean line). The dashed line at y = 1 represents the 1:1 proportion, where the treatment and control are the same.

Timing of the peak (*t_max_*) and minimum (*t_min_*) cortisol concentrations were estimated as derived parameters by setting the first derivative of the cubic function to zero and solving for time. Peak cortisol concentration (*cort_max_*) was estimated by back substituting *t_max_* into the first derivative equation. Distribution time was estimated as equivalent to *t_max_*; elimination time was estimated as the difference between *t_min_* and *t_max_*. Models were fit separately for each experiment (ACTH or cortisol injections), matrix type, and treatment group (Figure 1). Models were also fit for control groups, with matrix types (plasma, or mucus) pooled across experiments. Uncertainty in the derived timing and cortisol concentration parameters were expressed using a standard 90% credible interval (CI). Analyses of plasma cortisol levels were constrained to the first sampling window (0–12 hours) to account for repeat sampling, whereas analyses of mucus cortisol levels were conducted over the entire experimental timeframe (0–84 hours). Final peak identification and characterization was accomplished by dividing each treatment model curve by its corresponding control model curve (e.g. plasma ACTH curves divided by plasma control curves) to obtain a proportional curve (Figure 2). These resulting proportional curves helped elucidate the total impact of the treatment as compared to background effects of handling. Significance of *cort_max_* relative to two baseline thresholds—mean time 0 values and temporally aligned modelled control values—was calculated as the proportion of MCMC samples where *cort_max_* was greater than each threshold.

## Results

### ACTH administration

Estimated peak cortisol concentrations following administration of ACTH differed in both timing and magnitude between matrices (Figure 1). Model curves for the ACTH treatment show peaks in plasma cortisol concentration at 2.86 hours (90% CI = 0.42–8.06 h) and at approximately 12 hours (Figure 1). Note, our study design and results failed to capture the full peak resulting from the rise in cortisol concentration observed at 12 hours. Consequently, we present both the timing and the magnitude of this rise as conservative minimum values. When examined as a proportion of the control model, only the 12-hour peak persisted (Figure 2), suggesting that it takes approximately 12 hours for plasma cortisol to reach peak concentration following ACTH administration in the absence of handling stress. Indeed, 96.5% of MCMC samples for this peak were higher than the mean time 0 value and 90.0% were higher than temporally aligned modelled control values. Both model and proportional curves for the ACTH treatment show a peak in mucus cortisol concentration at 61.72 hours (90% CI = 55.57–66.83 h; Figures 1 and 2), with 94.2% of MCMC samples higher than the mean time 0 value and 89.5% higher than temporally aligned modelled control values. Cortisol concentrations at estimated peaks were substantially larger in plasma (476.53 ng/ml; 90% CI = 328.60–744.50 ng/ml) than in mucus (20.88 ng/ml; 90% CI = 15.03–27.57 ng/ml; Figure 1). The range in measured plasma cortisol concentrations (minimal 47.19 ng/ml at 12 h to maximal 1097.04 ng/ml at 12 h) was much wider than in mucus (0.29 ng/ml at 0 h to 37.14 ng/ml at 0 h). Mean plasma cortisol concentrations at the time of capture were much lower for all 20 fish ($\overline{x} \pm sd$; 51.02 $\pm$ 71.31 ng/ml) than mean plasma cortisol concentrations at the time of injection (*t* = 0; 231.44$\pm$ 96.42 ng/ml).

### Cortisol administration

Estimated cortisol elimination timing also differed between matrices (Figures 1 and 2). For the cortisol injection group, both model and proportional curves suggest an elimination time in plasma of approximately 7.38 hours (90% CI = 6.60–12.30 ng/ml). For mucus, modelled and proportional curves suggest an elimination time of approximately 51.12 hours (90% CI = 0.00–138.07 h; Figures 1 and 2). We note that these elimination times are based on *t_max_* values at 3.59 hours (90% CI = 3.13–5.10 h) and 28.76 hours (90% CI = 0.00–107.44 h) for plasma and mucus, respectively. While 100.0% of MCMC samples for *cort_max_* were higher than both control and mean t0 values for plasma, these values were substantially lower for mucus (55% and 29%, respectively). Similar to the ACTH results, cortisol concentrations at estimated peaks were substantially larger than in plasma (1283.93 ng/ml; 90% CI = 979.92–1445.01 ng/ml) than in mucus (23.4 ng/ml; 90% CI = 12.61–82.95 ng/ml). The range in measured plasma cortisol concentrations (155.35 ng/ml at 12 h to 1468.26 ng/ml at 4 h) was also substantially greater than the range in measured mucus cortisol concentrations (4.38 ng/ml at 36 h to 76.84 ng/ml at 8 h). Again, plasma cortisol levels at the time of capture ($\overline{x} \pm sd$; 51.02 $\pm$ 71.31 ng/ml) were much lower than plasma cortisol levels at the time of injection (cortisol group, *t* = 0: 418.12 ng/ml; control, *t* = 0: 461.18 ng/ml).

## Discussion

By inducing cortisol synthesis and distribution using ACTH injections and inducing cortisol elimination using cortisol injections, we examined cortisol dynamics in Pacific halibut as reflected in both plasma and epidermal mucus by mimicking the stress response. Our results suggest that a multi-tissue approach to cortisol analysis allows for potential temporal stratification in application and interpretation from a single sampling event. Differences in the relative rates with which cortisol is distributed to (following stimulation of cortisol synthesis by ACTH injections) and eliminated (following exogenous cortisol injections) from plasma and mucus demonstrate unique temporal windows through which cortisol concentrations could be viewed and compared. In the following sections, we explore potential mechanisms leading to the observed disparity in cortisol distribution between plasma and epidermal mucus, consider the implications of our results on understanding stress response in Pacific halibut, and investigate the potential applications for multi-tissue approaches in applied fisheries research and management.

The stress response of vertebrates is an intricate system regulated at whole-body (hypothalamic–pituitary-interrenal axis; Wendelaar Bonga, 1997), local (paracrine signalling of adjacent cells; Bradford et al., 1992), and peripheral (caudal neurosecretory system; Lu et al., 2004) levels. This complexity, coupled with the potentially cross-reactive effects of endogenous and exogenous hormones, the relative amount of binding proteins in blood, the density of target tissue receptors, rate of tissue uptake, hepatic metabolic activity, and the catabolic rate of cortisol (Vijayan and Leatherland, 1990; Mommsen *et al.*, 1999), complicates the interpretation of induced cortisol peaks. Paracrine, or ultra-short-loop feedback effects, have also been found to have attenuating effects on cortisol secretion rates after exogenous ACTH injections in brook charr (*Salvelinus fontinalis*) and coho salmon (*Oncorhynchus kisutch*), resulting in lower cortisol secretion rates that varied by dose (Vijayan and Leatherland, 1990; Bradford *et al.*, 1992). In addition, the interpretation of cortisol levels is context-dependent, varying by parameters such as season, sex, and age (Baker *et al.*, 2013a; Breuner *et al.*, 2008). Though further research on underlying physiological mechanisms and inter-individual variability is necessary, a foundational understanding of cortisol kinetics, specific to the species of interest, is a crucial first step to understanding the scale of the cortisol response to stress. As a result of this complexity, assessing cortisol kinetics by inspecting induced cortisol peaks is challenging, and we confine our interpretation to an exploration of the differences in the relative timing and magnitudes at which cortisol concentrations peaked and declined in our two study matrices.

Matrix-specific variation in the timing at which cortisol concentrations reached peak values after inducing cortisol synthesis by ACTH administration can largely be attributed to differences in the complexity of the pathways by which cortisol is distributed to plasma versus epidermal mucus. In the case of plasma, the route is comparatively direct. Upon reception of ACTH, interrenal tissue releases cortisol directly into the blood for distribution to target tissues (Romero and Butler, 2007; Touma and Palme, 2005; Mommsen *et al.*, 1999; Wendelaar Bonga, 1997). In contrast, cortisol reaches epidermal mucus by an indirect route involving excretion through the goblet cells (Shephard, 1994). The delay in peak cortisol concentration in epidermal mucus observed in our study (Figure 1) is likely the consequence of the time required for cortisol to reach the mucus through this indirect route. Guardiola *et al.* (2016) observed peak cortisol concentrations in gilthead seabream (*Sparus aurata L.*) plasma at 2 hours after exposure to acute crowding, similar to the timing of the initial plasma cortisol peak after ACTH treatment (2.9 h) observed in the present study. However, based on the contrast in the treatment and control groups we interpret the initial peak in plasma cortisol as an effect due to handling (Figure 2), and the rise at 12 hours to be the effect of cortisol production from ACTH injection. Mucus cortisol peaked at 24 hours in the experiment by Guardiola *et al.* (2016), similar to the distribution timing of mucus cortisol in our control group (19.2 h). The relative timing of peak concentrations described by Guardiola *et al.* (2016) was approximately 22 hours between peak values in plasma and mucus. However, we observed a much longer delay of approximately 50 hours between peak values in plasma and mucus in our ACTH treatment group, possibly due to differences in fish size, holding conditions, or species-specific differences between the two studies. Despite this contrast, the temporal difference in peak cortisol levels between plasma and mucus shown in our results offers the possibility of examining physiological stress responses at different time scales.

Differences in the peak concentration values of cortisol between plasma and epidermal mucus also reflected disparities in the routes by which cortisol reaches the two matrices, specifically suggesting that the movement of cortisol is constrained during the journey from plasma to mucus (Figure 1). Noticeable depreciations in cortisol concentration between the two matrices are likely the result of decreases in cortisol caused by a combination of cortisol metabolism and passive leaking into goblet cells following the release of cortisol from the interrenal gland into the bloodstream. As such, epidermal mucus acts as only one of several pathways by which cortisol is discarded from the body. Our results parallel those of Simontacchi *et al.* (2008), who noted higher cortisol concentrations in the plasma of sea bass (*Cyprinus carpio*) compared to mucus. Similarly, Guardiola *et al.* (2016) found that peak cortisol concentrations in gilthead seabream (*Sparus aurata L.*) were approximately 3 times higher in plasma than in epidermal mucus after exposure to acute crowding. While peak plasma cortisol concentration estimates in our study were generally higher than those observed in previous studies (Haukenes and Buck, 2006; Bertotto *et al.*, 2010; Guardiola *et al.*, 2016), we attribute this difference to the use of chemical cortisol induction (ACTH) rather than a physical stressor and, potentially, the effects of captivity and handling stress on our subjects. We also interpret the higher cortisol levels at the beginning of the cortisol administration experiment to be an indication of higher stress levels due to prolonged captive conditions and increased handling relative to the beginning of the ACTH experiment. While we do not expect that stress levels of wild-caught adult halibut engaged in captive research will ever return to pre-capture levels; by presenting the results from our treatment groups as a proportion of our control groups, we aim to demonstrate the effect of ACTH and cortisol treatments independent of stress associated with capture and holding. Further work with juvenile and adult halibut held long-term in captivity, and the examination of changes in acclimation time, would provide additional relevance for future work in stress physiology. However, we note that the mucus cortisol concentration values observed in our study were similar to those reported by previous authors (Guardiola *et al.*, 2016; Mercado *et al.*, 2016), and that the cortisol response can vary widely among species, environmental conditions (e.g. temperature, seasonality; Pankhurst, 2011), and other biological parameters (e.g. sex, reproductive maturity, age; (Barcellos *et al.*, 2012; Baker *et al.*, 2013b).

Unlike the ACTH experiment, in which we observed strong transfer of cortisol from plasma to mucus, results from the cortisol experiment suggest relatively little transfer between the two matrices (Figure 2), potentially reflecting differential effects of endogenous and exogenous cortisol. For example, Foster and Moon (1986) observed that administrations of exogenous cortisol resulted in an increase in plasma cortisol metabolism and a concomitant decrease in the synthesis of endogenous cortisol in American eels (*Anguilla rostrata*). In our study, similar effects caused by exogenous cortisol administration may have artificially increased plasma elimination times, and functionally limited cortisol transfer between plasma and mucus. Consequently, we 1) interpret the plasma elimination times presented here as a conservative underestimate, 2) recommend that future studies consider adopting a two-compartment model (*sensu lato*  Geisz and Bourin, 1986) when attempting to study cortisol kinetics using exogenous administrations, and 3) encourage consideration of treatment protocol (endogenous vs exogenous), and physical and environmental stress when evaluating the transfer of cortisol between plasma and mucus.

The precise application of a comparative multi-tissue approach to cortisol analysis in Pacific halibut is limited by our current understanding of the specific physiological mechanisms governing cortisol kinetics among tissues including dose effects, the roles of binding proteins, glucocorticoid receptors, hepatic metabolism, and cross-reactive effects from other hormones (Vijayan and Leatherland, 1990; Mommsen *et al.*, 1999; Philip and Vijayan, 2015; Liu *et al.*, 2016; Aerts, 2018). However, our results show both distinct separation in the timing at which cortisol reached peak concentrations following induction in plasma and mucus, and that cortisol levels in mucus are generally reflective of changes in plasma. This suggests the potential for temporal stratification among sampling tissues leading to a multi-tissue approach to cortisol analysis in teleost fish like Pacific halibut.

The potential for multi-tissue cortisol analysis has substantial implications for monitoring and assessment in fisheries management and aquaculture. For example, comparative analysis of plasma and mucus cortisol levels can be used as an indicator of the relative physiological states of non-target fish before and after capture by fishing vessels. In this case, if a Pacific halibut were captured on a trawling vessel, sampled, and then discarded alive at sea, we would expect that plasma cortisol levels would reflect the stress of the capture, whereas mucus cortisol levels would reflect basal stress levels prior to capture. This information may assist agencies and regional fisheries management organizations such as the International Pacific Halibut Commission with assessing or improving discard mortality estimates, furthering their ability to sustainably manage this species. Likewise, multi-tissue cortisol analysis may be used to provide information for fish before and after stocking in aquaculture facilities without causing additional stress by resampling. We recommend conservative application of the use of mucus as part of a multi-tissue approach to cortisol analysis concurrent to investigations into physiological mechanisms influencing cortisol kinetics.

## Funding

This work was supported by the National Oceanic and Atmospheric Administration Saltonstall-Kennedy Grant Program (award number NA17NMF4270240) and by Alaska Education Tax Credit funds contributed by the Groundfish Forum.
